# Investigating the system effect of reporting multidisciplinary care measures for cancer services in New South Wales, Australia

**DOI:** 10.1186/s12913-021-07050-7

**Published:** 2021-10-02

**Authors:** Kahren M. White, Ru K. Kwedza, Holly Seale, Reema Harrison

**Affiliations:** 1 School of Population Health, University of New South Wales, Sydney, Australia; 2 Cancer Institute NSW, Locked Bag 2030, St Leonards, NSW 1590 Australia

**Keywords:** Multidisciplinary teams, Cancer, Key performance indicators, System improvement, Quality improvement

## Abstract

**Background:**

Multidisciplinary cancer care to facilitate the provision of patient centred and evidence-based care is considered best practice internationally. In 2016 multidisciplinary care measures were developed for all local health districts across NSW. The aim of this study was to identify system-level changes and quality improvement activities across the NSW cancer system linked to reporting on these measures.

**Methods:**

Focus group discussions were used to generate a synergy of ideas from key stakeholders. An exploratory descriptive approach was used within the ontological position of Framework Analysis, the analysis method chosen for this research study, sitting most closely within pragmatism. The use of Framework Analysis in the analytic strategy is because it is well-suited to addressing policy issues and maintaining specific focus within a wider dataset.

**Results:**

Two focus groups were held with a total of 18 purposively selected participants. Four primary themes emerged: value of electronic documentation; role clarity; relationships; and future development of measures. Key findings included that the reporting of performance measures has expedited the development of electronic documentation and data extraction from the multidisciplinary team meeting (MDT), identified barriers and facilitators to MDT data collection and supported MDT improvement activities across NSW.

**Conclusions:**

The findings of this study have highlighted that MDTs and their meetings across NSW are harnessing technological advancements to support and further develop their MDTs, as well as the challenges of implementing new processes within the MDTM. This study adds a unique contribution to knowledge of how the reporting of measures can assist in understanding variation in the development and implementation of multidisciplinary teams, as well as highlighting future programs of work to decrease variation in multidisciplinary team meetings and quality improvement activities.

**Supplementary Information:**

The online version contains supplementary material available at 10.1186/s12913-021-07050-7.

## Background

Multidisciplinary cancer care is considered best practice internationally, facilitating the provision of patient centred and evidence-based care [1]. Multidisciplinary cancer care includes input from cancer-specific multidisciplinary team meetings (MDTMs) to facilitate multidisciplinary discussion and decision making for diagnosis, staging and initial treatment planning for people with a suspected or confirmed cancer diagnosis. The use of MDTMs has been identified as being essential to ensure the provision of evidence based and high quality care to patients as part of these recommendations [2]. Following the systemwide implementation of MDTMs in the United Kingdom from the mid 1990’s the National Breast Cancer Centre in Australia initiated a national multidisciplinary care demonstration project for breast cancer in 1999, to examine the impact, cost and acceptability of implementing multidisciplinary care for women with breast cancer across Australia [3]. Following this successful demonstration project, multidisciplinary care and MDTMs were increasingly implemented across Australia and have now become standard practice nationally [4–6].

Multidisciplinary care and the use of MDTMs provides a systems-level approach to improve adherence to clinical guidelines, as well as improving the team approach to the diagnosis, staging and recommended treatment for people with a cancer diagnosis [7]. Variation from clinical guidelines or the established clinical evidence base may be recommended by the treating clinician, discussed and agreed on as appropriate in the MDTM, given the patients preferences, performance status and comorbid disease. MDTMs also have a role in clinical governance, ensuring evidence based treatment decisions are made for those patients discussed [7].

Key performance indicators and measures have been developed across a number of jurisdictions, both nationally and internationally, to monitor the development and improvement of multidisciplinary teams and their meetings. Key performance indicators are used for continuous performance monitoring and assisting in focusing quality improvement programs [8]. The focus of many of these indicators are on the structure and process of the MDTM, as opposed to the outcomes of the MDTM, which may include how the team functions as a cohesive team, as well as its role in staging and treatment planning for people with a suspected or confirmed diagnosis of cancer. Supporting the rationale for this approach were the findings from Gort et al. who investigated how multidisciplinary breast cancer teams in the Netherlands applied process, structure and outcome indicators as part of their multidisciplinary teams quality improvement [9]. They identified the importance of both the controllability of the measure, as well as the actionability of indicators, being key in understanding if indicator use in a multidisciplinary team context lead to improvements [9].

The Cancer Institute New South Wales is the cancer control agency for the state of New South Wales (NSW) in Australia. The key goals and objectives of the Cancer Institute NSW are to reduce the incidence of cancer, increase the survival of people with cancer, and improve the quality of life for people living with cancer across NSW [10]. A prioritised action of the current NSW Cancer Plan is that all people diagnosed with cancer have their care overseen by a multidisciplinary team [10]. To monitor the ongoing implementation of multidisciplinary cancer care and the improvement in multidisciplinary processes the Cancer Institute NSW developed a system-level quality improvement program to develop multidisciplinary care measures as a way to harmonise and reduce variation in multidisciplinary care practice. Six multidisciplinary care measures were implemented from 2016 to 2018 (Table 1), in line with the national and international evidence base for multidisciplinary care and multidisciplinary team indicators [7, 11, 12].

**Table 1:** Multidisciplinary care measures for NSW

Measure financial year 2016/17	
1	Number of patients with cancer that have their care overseen by an MDT by stage of disease, for lung, ovarian, rectal and liver cancer.
2	Number of patients with lung cancer that have their care overseen by an MDT prior to commencement of any treatment modality by stage of disease.
3	Number of times that Medicare Benefits Schedule (MBS) billing has occurred to support MDT activity.
4	Proportion of patients for whom documentation of their planned treatment is sent to the patients’ General Practitioner (GP) within 7 days of MDT/or any other time measure point along the treatment pathway.
Measure financial year 2017/18 and 18/19	
1	Proportion of multidisciplinary teams listed on Canrefer within the Local Health District that have data updated every 6 months within 4 weeks of receipt of request to update.
2	Number of new primary lung cancer patients by stage of disease that are presented at an MDT prior to commencement of any treatment modality.
3	Proportion of MDTs using MBS billing in the Local Health District.
4	Local Health District capability for MBS billing for MDTs.
5	Proportion of patients presented at MDT where a discussion summary is then sent to the GP.
6	Median timeframe from MDT to GP communication.

To date, knowledge of the use of multidisciplinary care measures across NSW, Australian and international cancer services, including the resulting process and/or quality improvement activities, are unknown. The aims of this study were therefore to:

- explore how cancer services in NSW have interpreted and collected the multidisciplinary care measures;
- identify system-level changes and quality improvement activities initiated in the NSW cancer system that may have occurred as a result of reporting on the multidisciplinary care measures; and
- explore key stakeholder perceptions of the multidisciplinary care measures that still require development.

## Methods

A qualitative methodology was identified as being the most appropriate to answer the research questions. Through the use of a focus group method, we were able to explore how the key stakeholders who participated thought and felt about the development, collection and reporting of the multidisciplinary care measures, as well as discuss any quality improvement activities initiated as a result of reporting on the multidisciplinary care measures [13]. One of the strengths identified when choosing the focus group method for this study was the ability to use the homogeneity of the focus group members as a positive and the synergy of ideas resulting from the group method discussion [13, 14]. However, there was representation from metropolitan and non-metropolitan local health districts, as well as different ages and genders of participants. Ethics approval for this study was granted by the UNSW Human Research Ethics Advisory Panel G (Health, Medical, Community and Social) in July 2018, approval number HC180404. All participants provided written informed consent to participate in the research study. The participant information sheet is available as Additional file 1: Appendix A.

### Sample

The participants were key stakeholders of the Cancer Institute NSW that had previously participated in the development of the multidisciplinary care measures, and also held responsibly in providing the Cancer Institute NSW with data on the multidisciplinary care measures every 6 months. Three key stakeholder roles were identified: Cancer System Innovation Managers; Directors of Cancer Services and Innovation (Rural); and NSW Cancer Registry Program Managers. These roles share similar experiences across the NSW cancer system. There were participants from ten of the fifteen local health districts and one speciality health network providing adult cancer services in NSW. One local health district does not receive funding for multidisciplinary care, as they do not hold local MDTMs. This local health district was excluded from the study, with fourteen local health districts and one speciality health network being eligible to participate in the study.

### Procedure

Data were collected from two focus groups held face to face at the Cancer Institute NSW offices, with key stakeholders from across fourteen NSW local health districts and one speciality network. Stakeholders were invited to participate in the focus groups through an initial email from HS, with the participant information sheet which included information on the research. This was to ensure participants did not feel pressure to participate in the research, as KW had pre-existing professional relationships with the stakeholders through her role at the Cancer Institute NSW. Participants provided written consent to participate in the study. A discussion guide was developed by KW and reviewed by RK, HS and RH (Additional file 2: Appendix B). The discussion guide was pre-tested with four staff of the Cancer Institute NSW prior to the focus groups, to ensure acceptability of questions and that the questions were understandable and elicited responses relevant to the study aims. The Cancer Institute NSW staff (involved in pre-testing) were chosen as they all had experience working in strategic positions in local health districts prior to working at the Cancer Institute NSW, aligned to the experience of the focus group sample. These Cancer Institute NSW staff were not included in the main study. Focus groups lasted up to 90 min in duration. Both focus groups were facilitated by KW, as well as being audio recorded and transcribed by KW.

### Analysis

Analysis was undertaken using thematic content analysis within the structure of the Framework Analysis method developed by Ritchie and Spencer [15]. Framework Analysis was developed to support the analysis of qualitative applied policy research and involves a process of familiarisation with the content, identification of a thematic framework, indexing of the themes, charting the themes, and mapping and interpretation of the themes [16].

Member checking was conducted during the focus groups to ensure credibility of the data, and to ensure KW accurately understood the participant’s views. The recordings of the two focus groups were listened to multiple times, to assist in understanding the context of the discussion. Further familiarisation of the transcripts was undertaken with repeated reading and notes taken on initial themes. An initial thematic framework was developed, indexed and sorted into the transcripts of the focus groups, using NVIVO 12 (QSR International Pty Ltd., Version 12, 2018) for management of the data. Further indexing of the data was undertaken, identifying key themes and sub-themes that fit under them. Data extracts were then reviewed, allowing for refinement of the sub-themes and ensuring themes were not missing from the developing framework. The final stage of framework analysis was to develop a set of matrices linking the themes, sub-themes and allocated statements from the focus group participants. This process allowed for a final review of the themes and to ensure participant statements are fully captured in the analysis. KW developed initial codes which were then discussed with HS and RH to reach a consensus.

## Results

Two focus groups were undertaken with demographic information presented in Table 2. Twenty-five participants were eligible and invited to participate in the study, with 18 people participating (72% participation rate).

**Table 2:** Characteristics of the study cohort

	Focus group 1 (N)	Focus group 2 (N)
Participants	7	11
Geographical area		
Metropolitan local health district	4	6
Regional local health district	3	5
Local health districts	7	10
Sex		
Male	1	2
Female	6	9

Four primary themes emerged from the data: harnessing technological advancements; role ambiguity in documentation; clinical relationships within the multidisciplinary team; and linking multidisciplinary teams, funding and performance outcomes.

### Harnessing technological advancements

Most participants reported that in the past 2 years there has been effort by both the Cancer Institute NSW and cancer services on understanding how the Oncology Medical Information System (OMIS) and other electronic medical records were being used to develop agendas and document the MDTM discussion, as well as how they could be further developed for this purpose. There are a number of electronic medical record systems used across the cancer system in NSW. These include MOSAIQ®, ARIA, CHARM™ and Cerner PowerChart. NSW Health does not prescribe the electronic system used for documenting from the MDTM, but there is an expectation that the discussion and decisions made are documented. Further development has been through the electronic systems allowing pathology and radiological images to be viewed in the meeting, as well as real time documentation to be completed. Some participants highlighted that where a cancer service did not have an electronic medical record in place they needed to implement and develop these capabilities. Through the development of the OMIS and setting up electronic data extraction a number of participants across all types of key stakeholders found they were able to extract data more efficiently. They reported this has assisted in their ability to provide data on the multidisciplinary care measures, as well as answer questions and provide reports for their local clinicians. As one participant stated:

[we are] behind the 8 ball really where we have had all of these different databases collecting MDT dates, basically dates of presentation, they have lots of paper, so I think it really highlighted to us that [electronic] templates need to be developed. (Participant 5)

It became clear during both focus group that there is diversity in how each MDTM across NSW, both within and between local health districts, choose to use the OMIS system. This can lead to challenges in the ability to extract data in a consistent format. As one participant stated:

[We are] on our way to having all of our MDT’s electronically capture data in a way that is useful for registry data, as well as their own research. (Participant 1)

However, a number of participants highlighted the need to allow flexibility in how each MDTM interacts with and utilises the OMIS system. There is a risk in standardising all MDTMs to function in the same way that the innovation and flexibility that may lead to a specific MDTM working in a way that is sustainable and effective for them may be lost.

As one participant stated:

The only thing there is when they do change over to using the source system [OMIS], each group will decide they might want to use it quite differently to the other group, some may use questionnaires, and purely putting their data in their questionnaires but that is not really populating the source system data fields, but then some choose to directly use the data fields that are already in the source system and will just do the extra bits in a questionnaire. There is not a one-size-fits-all solution there (Participant 3).

### Role ambiguity in documentation

Participants discussed the challenge of role clarity, specifically relating to the responsibility for documentation during the MDTM. They discussed the challenges of how to implement documentation if it was not already occurring, as well as supporting clinicians to understand that the information they input at the MDTM can be used as data to answer quality improvement and research questions. Several challenges were highlighted by participants regarding changes to the way documentation had traditionally occurred within the MDTM. Firstly, the issue of responsibility was raised, where some participants requested by clinicians to have administrative staff or data managers document in the MDTM, where other participants reported their advanced trainees and MDTM chairs take responsibility for documentation. As one participant stated:

[MDT chairs] are all asking for data managers, they all ask for someone to put their data in for them. My take has been the registrars should be doing it, you should have a scribe … no one sees it as their, you know, their role, to actually enter data (Participant 1).

As another participant stated:

The registrars take that role [of documentation] and at the end of that patient discussion, the end of meeting discussion and clarification is checked with the chair, can I just confirm you said blah blah, and it's done [approved] immediately. (Participant 13).

A few participants felt the responsibility for documentation should sit with clinicians for legal reasons, as clinical decisions are being recorded, which is out of the scope of responsibility of an administrative staff member or data manager. Participants discussed the challenges that arose when roles in the MDTM were not clear, with members not understanding the purpose or need to document the MDTM discussion and clinical recommendations or decisions made. As the MDTM discussion is a clinical discussion with clinical decisions being made, there is a legal requirement to ensure both the discussion and recommendations are documented in the patient’s medical record (46). As one participant stated:

[MDT chairs] are all asking for data managers, they all ask for someone to put their data in for them. My take has been the registrars should be doing it, you should have a scribe … .no one sees it as their, you know, their role, to actually enter data. (Participant 1)

### Clinical relationships within the multidisciplinary team

The impact relationships had on their ability to collect the multidisciplinary care measures over time was discussed by participants, as well as clinician and executive level buy in for support of the multidisciplinary team and its developing processes. The development of key relationships with those clinicians who take on the role of clinical champion within the multidisciplinary team has led to improvements in both the MDTM process, as well as documentation. This included the development of referral processes and documentation, initiation of real time documentation in the MDTM and the development of MDTM summaries being sent to General Practitioners. Participants highlighted the need to link documentation to improving clinical processes, with this giving the clinicians an understanding on why they should be documenting. As one participant stated:

The young guys who are really keen they want to do this, and they want their data and they want to be able to show what they’ve done. To them it’s just what they should be doing. (Participant 12)

Participants also described the challenge in making changes within the multidisciplinary team and MDTM. Clinicians resistance to taking up live documentation during the MDTM appears to stem from a perception that this is not their role, and appears to lead to a perceived barrier to change within the MDTM.

### Linking multidisciplinary teams, funding and performance outcomes

One of the aims of the focus groups was to discuss the current multidisciplinary care measures being collected, as well as elicit ideas from the group on future measures that could be collected; specifically, measures that are meaningful to the ongoing development of MDTs and their meetings across NSW. Both the benefits and challenges of the multidisciplinary care measures, and the need to provide data every 6 months to the Cancer Institute NSW were highlighted by participants. There was limited understanding across the NSW cancer system of the link between these measures and the provision of funding to support multidisciplinary cancer care across NSW. A number of proposals were made for future measures that were considered more meaningful than the current measures. Commonly supported ideas included the development and implementation of criteria for patient discussion at the MDTM, expanding reporting to more cancer types and improved linking of the measures to outcomes. Through linking future measures to clinical outcomes, the measures may be able to mature from being limited to process measures, such as the number of cases presented at an MDTM, to providing more meaningful information on the role of the MDT and its meeting. As one participant stated:

I guess the first question is do you have criteria, second question is how close are they to guidelines, are there any guidelines, and is it is based on the particular resourcing of the facility that they’re not following guidelines? (Participant 16)

## Discussion

The three aims of this study were to explore how cancer services across NSW have interpreted and collected the multidisciplinary care measures; to identify system level changes and quality improvement activities that have been initiated as a result of the multidisciplinary care measures; and to explore key stakeholder perceptions of measures that require development into the future. Four primary themes emerged from the data: harnessing technological advancements; role ambiguity in documentation; clinical relationships within the multidisciplinary team; and linking multidisciplinary teams, funding and performance outcomes.

These findings indicate that the implementation of the multidisciplinary care measures has led to a range of changes in the way that MDT activity is documented, including greater use of technology, particularly in documentation of discussion and decisions, as well as further development of processes to support the MDT. Study participants recommended the development of further measures that include the measurement of outcomes from the MDTM. This development will assist in measuring outcomes linked to the oversight of people with a diagnosis of cancer by an MDT, as this has become best practice internationally and is a priority action of the NSW Cancer Plan [10, 17].

One of the key developments over the past 10 years in health services has been the development of electronic medical records. The findings of this study highlight how cancer services, and more specifically MDTMs, have harnessed these advances in technology, through allowing radiology and pathology to be reviewed electronically at the MDTM, as well as to facilitate live documentation at the MDTM into the OMIS, with some teams having developed the ability for summary letters to be sent automatically to the General Practitioner following the MDTM.

The findings of this study reflect existing Australian studies that have investigated the use of information communication technologies in MDTMs (49). A study by Janssen et al. found that data collection in the MDTM and use of electronic medical records was inconsistent, with only one MDTM in their study cohort documenting the MDTM discussion in an electronic medical record or MDTM database in real time (49). With the work being undertaken in NSW to ensure all cancer services have an OMIS, primarily to ensure the electronic prescription of systemic therapy medications, with a secondary aim of facilitating electronic documentation across the cancer service, including the MDTM, as well as to enable electronic extraction of data into the NSW Cancer Registry, the current study indicates much has changed across NSW since Janssen’s study in 2014 (49).

Many MDTMs are documenting the discussion in real time, and for those services where this has not occurred, there is a focus on embedding real-time documentation and improving role clarity around this. Documentation in real time during the discussion is recommended across many jurisdictions, both nationally and internationally (19, 22, 50, 51). The results of the current study demonstrated the different levels of capability across NSW of documentation during the MDTM. The findings highlighted the innovation in MDTMs in how positions are used to support the needs of the team, as well as the increasing development and use of the electronic medical record system to support live documentation. One of the challenges observed in the focus group discussion was in relation to role clarity and the understanding of language and how it is used. When the word ‘data’ is used in relation to documentation in the OMIS participants reported clinicians did not perceive this as their responsibility. When discussing clinical documentation as part of standard clinical practice the participants reported the clinicians were more likely to take on responsibility for documentation at the meeting.

Challenges related to documentation at the MDTM and the provision of discussion summaries to the General Practitioner often have a base in medicolegal concerns from clinicians. Participants reported that some clinicians are reluctant to document the group discussion and decisions at the MDTM due to the concern that from a legal perspective they may be ‘locked into’ the treatment plan made at that time. One of the early journal papers highlighting the medicolegal implications of group decision making in cancer MDTMs was from Sidhom & Poulsen (52). They highlighted the group decision making process of MDTMs, where clinicians are involved in the discussion and recommendation of treatment for patients they may never meet or treat, and the legal ramifications if a patient later sues for medical negligence (52). Sidhom & Poulsen investigated this further by completing a study to investigate the awareness of doctors who participated in MDTMs of their personal responsibility and liability for decisions made within the group context of an MDTM (46). This study demonstrated that doctors had limited awareness of their legal responsibility for decision making within the MDTM, also highlighting challenges with the culture of MDTMs, leading to doctors feeling that they were unable to formally dissent on treatment decisions being made at the meeting (46). The authors go on to recommend ways to improve documentation through educating doctors on their medicolegal responsibilities within the MDTM, ensuring MDTMs are adequately resourced to support the technology required in the meeting, enabling detailed recording of the MDTM discussion from both the group and individual clinician perspectives, and improving patient involvement in decision making about their treatment (46).

The findings of our study identified that it is not always a clinician who documented the MDTM discussion, with extra resources often being requested to support MDTM documentation. A recent study by Rankin et al. investigated MDTMs across different settings to understand how evidence informed the MDTM [17]. The study identified a number of different multidisciplinary team members being responsible for meeting documentation, including the multidisciplinary team coordinator (often an administrative role), multidisciplinary team clinical lead, registrar and nurses [17]. This was also reflected in the finding of our study. In the Rankin et al. study only a third of MDTMs were providing MDTM treatment summaries to the patients General Practitioner, counter to the recommendations set out in the Australian national optimal care pathways and by Cancer Australia [5, 17, 18]. The challenges of implementing General Practitioner MDTM discussion summaries was highlighted in the current study, with participants reporting the difficulty they faced in implementing this as a standard process from the MDTM, due to the lack of support from clinicians, despite this being currently recognised as best practice.

The primary limitations to this study include that not all of the local health districts in NSW who received funding at the time for multidisciplinary care were represented in the focus group participants, leading to experiences from those districts to be missing from the data. There is also disparity between the electronic capabilities of each local health district. This study also provided only a limited view of the NSW cancer system in the focus groups, due the focus on the key stakeholders invited to participate, leading to the voice of clinicians and consumers being absent from this research. There are limitations to using focus groups as a method of data collection, such as limited active participation and discussion with group members, barriers to interaction in a group setting, limited depth in the discussion, leading to limitations in understanding participants experiences and how these meet the research aims [14]. Through the use of a topic guide and clear facilitation some of these limitations may have been able to be limited. One of the key strengths of using a focus group method is how group interaction can support information being discussed that may not have been expected by the researcher, as well as providing and open and supportive environment for discussion [14].

## Conclusion

The findings of this study have highlighted that MDTs and their meetings in a number of local health districts in NSW are using the advances in information technology to support and further develop their MDTs, as well as the challenges of implementing new processes within the MDTM. Participants were able to discuss the utility of the previous measures and discuss measures that may be developed for the future to continue to support quality improvement activities across their cancer services. There remain areas of focus for improving both the process and function of MDTs and their meetings in a number of local health districts in NSW, including documentation at the time of the MDTM discussion, developing electronic processes to improve both the running of the MDT meeting, as well as the ability for data to be extracted directly from the source system to the NSW Cancer Registry. These improvements in how MDTMs function may be able to improve unwarranted clinical variation, through ensuring increased numbers of people with a cancer diagnosis have their cancer care overseen by an MDT, with the aim of improving access to evidence-based treatment.

As more measures to monitor multidisciplinary cancer care across NSW are developed and implemented, it is important to continue to research the effect these measures have on the NSW cancer system. Next steps for this may include mapping the maturity in the use of information technology systems in all MDTMs in NSW and linking those teams who are mature in their use, with those who are in the early stages of implementation, as well as improved clarity in MDTM governance at each local health district to improve the understanding and acceptance of roles and clinical relationships within the MDTM. Once the system has reached a level of maturity where outcome measures are able to be reported, it will be important to report the effect that these types of measures have on the cancer system, and ultimately on improving outcomes for people living with cancer across NSW.

## Supplementary Information

**Additional file 1.**

**Additional file 2.**

## Data Availability

The deidentified data used and analysed during the current study are available from the corresponding author on reasonable request.

## Abbreviations

MDT: Multidisciplinary team
MDTM: Multidisciplinary team meeting NSW New South Wales
OMIS: Oncology medical information system
