# Supplementary material for: Investigating the system effect of reporting multidisciplinary care measures for cancer services in New South Wales, Australia
Source: BMC Health Serv Res. 2021 Oct 2;21:1044. doi: 10.1186/s12913-021-07050-7 (PMC8487574; doi:10.1186/s12913-021-07050-7)
Supplement: Supplementary file 1 — Additional file 1. [file 12913_2021_7050_MOESM1_ESM.docx]

1. **What is the research study about?**

You are invited to take part in this research study. The research study aims to explore how cancer services in NSW have used the collection and reporting of multidisciplinary care measures, and the specific impact this has had on local cancer services. You have been invited because you are a Cancer System Innovation Manager, Director of Cancer Services and Innovation (Rural) or a NSW Cancer Registry Program Manager.

1. **Who is conducting this research?**

The study is being carried out by the following researchers: Kahren White, Project Manager Quality and System Performance, Cancer Institute NSW; Dr Holly Seale, Senior Lecturer, School of Public and Community Medicine, University of New South Wales; Dr Reema Harrison, Senior Lecturer, School of Public and Community Medicine, University of New South Wales. This study is being undertaken as part of Ms White’s Doctor of Public Health, University of New South Wales.

1. **Inclusion/Exclusion Criteria**

Before you decide to participate in this research study, we need to ensure that it is ok for you to take part. The research study is looking recruit people who meet the following criteria: Cancer System Innovation Managers, Director of Cancer Services and Innovation (Rural) and NSW Cancer Registry Program Managers.

1. **Do I have to take part in this research study?**

Participation in this research study is voluntary. If you do not want to take part, you do not have to. If you decide to take part and later change your mind, you are free to withdraw from the study at any stage.

If you decide you want to take part in the research study, you will be asked to:

- Read the information carefully (ask questions if necessary);
- Sign and return the consent form if you decide to participate in the study;
- Take a copy of this form with you to keep.

1. **What does participation in this research require, and are there any risks involved?**

If you decide to take part in the research study, you will be asked to participate in a face to face **focus group**. You will be asked questions about the collection of the multidisciplinary care measure data and any impact reporting on these measures has had in your cancer centre. It should take approximately 90minutes to complete. Following the focus group you will be asked to complete a short electronic survey to collect demographic information about the study participants.

Each focus group will be held at the Cancer Institute NSW, Eveleigh NSW. There will be one of two sessions offered to the Cancer System Innovation Managers, Director of Cancer Services and Innovation (Rural). One group will be held in the last 90 mins of the regular face-to-face meeting for rural and regional members. A second group will be held at a convenient time for metropolitan Cancer System Innovation Managers, with a consensus time and date set up via a doodle poll. The NSW Cancer Registry Program Managers group will be held in a 90-minute session as part of their monthly face to face meetings held at the Cancer Institute NSW.

To ensure we collect the responses accurately, we seek your permission to digitally record the focus group audio using an audio recorder. These recordings will be transcribed by the student investigator, Kahren White. We don’t expect the questions to cause any harm or discomfort, however if you experience feelings of distress as a result of participation in this study you can let the research team know and they will provide you with assistance.

1. **What are the possible benefits to participation?**

We hope to use information we get from this research study to further refine the multidisciplinary care measures in future years and to understand the impact of the reporting of these measures on the NSW cancer system.

1. **What will happen to information about me?**

By signing the consent form you consent to the research team collecting and using information about you for the research study. Your data will be kept for a minimum of 7 years after the project’s completion. We will store information about you in a non-identifiable format on a secure server at the Cancer Institute NSW. Your information will only be used for the analysis for this research study, to identify the system impact of reporting multidisciplinary care measures for cancer services in NSW.

The information you provide is personal information for the purposes of the Privacy and Personal Information Protection Act 1998 (NSW). You have the right of access to personal information held about you by the University, the right to request correction and amendment of it, and the right to make a compliant about a breach of the Information Protection Principles as contained in the PPIP Act. Further information on how the University protects personal information is available in the [**UNSW Privacy Management Plan**](https://www.legal.unsw.edu.au/compliance/privacyhome.html).

1. **How and when will I find out what the results of the research study are?**

The research team intend to publish and/ report the results of the research study in a variety of ways. All information published will be done in a way that will not identify you. If you would like to receive a copy of the results you can let the research team know by email at kahren.white@health.nsw.gov.au*.*  We will only use these details to send you the results of the research.

1. **What if I want to withdraw from the research study?**

If you do consent to participate, you may withdraw at any time. You can do so by completing the ‘Withdrawal of Consent Form’ which is provided at the end of this document. Alternatively, you can ring the research team and tell them you no longer want to participate. Your decision not to participate or to withdraw from the study will not affect your relationship with UNSW Sydney or the Cancer Institute NSW.

If you decide to participate in the focus group, your comments along with other participants will be recorded during the group discussions. Because of the way in which the focus group discussions are recorded, the research team will not be able to withdraw or destroy individual participant responses.

1. **What should I do if I have further questions about my involvement in the research study?**

The person you may need to contact will depend on the nature of your query. If you require further information regarding this study or if you have any problems which may be related to your involvement in the study, you can contact the following member/s of the research team:

**Research Team Contact Details**

| **Name** | Holly Seale |
| --- | --- |
| **Position** | Senior Lecturer, School of Public Health and Community Medicine, University of New South Wales |
| **Telephone** | 9385 3129 |
| **Email** | [h.seale@unsw.edu.au](mailto:Kahren.white@cancerinstitute.org.au) |

**Support Services Contact Details**

If at any stage during the study you become distressed or require additional support from someone not involved in the research please call:

| **Name/Organisation** | Megan Varlow |
| --- | --- |
| **Position** | Manager Strategy and Operations, Cancer Institute NSW |
| **Telephone** | 8374 3624 |
| **Email** | [Megan.varlow@cancerinstitute.org.au](mailto:Megan.varlow@cancerinstitute.org.au) |

**What if I have a complaint or any concerns about the research study?**

If you have a complaint regarding any aspect of the study or the way it is being conducted, please contact the UNSW Human Ethics Coordinator:

**Complaints Contact**

| **Position** | UNSW Human Research Ethics Coordinator |
| --- | --- |
| **Telephone** | + 61 2 9385 6222 |
| **Email** | [humanethics@unsw.edu.au](mailto:humanethics@unsw.edu.au) |
| **HC Reference Number** | *HC180404* |

**Consent Form – Participant providing own consent**

**Declaration by the participant**

- I understand I am being asked to provide consent to participate in this research study;
- I have read the Participant Information Sheet or someone has read it to me in a language that I understand;
- I understand the purposes, study tasks and risks of the research described in the study;
- I understand that the research team will audio record the focus group; I agree to be recorded for this purpose.
- I provide my consent for the information collected about me to be used for the purpose of this research study only.
- I have had an opportunity to ask questions and I am satisfied with the answers I have received;
- I freely agree to participate in this research study as described and understand that I am free to withdraw at any time during the study and withdrawal will not affect my relationship with any of the named organisations and/or research team members;
- I would like to receive a copy of the study results via email or post, I have provided my details below and ask that they be used for this purpose only;

**Name: _____________________________________**

**Address: ___________________________________**

**Email Address: ______________________________**

- I understand that I a response email from the research will confirm my consent to participate in this research.

**Participant Signature**

| Name of Participant (please print) |  |
| --- | --- |
| Signature of Research Participant |  |
| Date |  |

**Declaration by Researcher***

- I have given a verbal explanation of the research study; its study activities and risks and I believe that the participant has understood that explanation.

**Researcher Signature***

| Name of Researcher (please print) |  |
| --- | --- |
| Signature of Researcher |  |
| Date |  |

**^+^An appropriately qualified member of the research team must provide the explanation of, and information concerning the research study.**

**Note: All parties signing the consent section must date their own signature.**

**Form for Withdrawal of Participation**

I wish to **WITHDRAW** my consent to participate in this research study described above and understand that such withdrawal **WILL NOT** affect my relationship with The University of New South Wales or the Cancer Institute NSW. In withdrawing my consent I would like any information which I have provided for the purpose of this research study withdrawn. I understand that the information collected about me during my participation in the focus group cannot be withdrawn given the nature of the focus group .

**Participant Signature**

| Name of Participant  (please print) |  |
| --- | --- |
| Signature of Research Participant |  |
| Date |  |

**The section for Withdrawal of Participation should be forwarded to:**

| CI Name: | Holly Seale |
| --- | --- |
| Email: | [h.seale@unsw.edu.au](mailto:Kahren.white@cancerinstitute.org.au) |
| Phone: | 9385 3129 |
| Postal Address: | School of Public Health and Community Medicine,  University of New South Wales  Sydney, NSW, 2052 |
