# Supplementary material for: Investigating the system effect of reporting multidisciplinary care measures for cancer services in New South Wales, Australia
Source: BMC Health Serv Res. 2021 Oct 2;21:1044. doi: 10.1186/s12913-021-07050-7 (PMC8487574; doi:10.1186/s12913-021-07050-7)
Supplement: Supplementary file 2 — Additional file 2. [file 12913_2021_7050_MOESM2_ESM.docx]

**Focus Group Discussion Guide: Investigating the system impact of reporting multidisciplinary care measures for cancer services in NSW**

**Introduction:**

In August 2016 and February 2017, the Cancer Institute NSW requested data for the first time on the multidisciplinary care measures outlined in the FY 2016/17 block funding agreements from each local health district that receives this funding. The Cancer Institute NSW then worked with you all on refining the multidisciplinary care measures for the FY 2017/18 block funding measures. Data were requested in August 2017 and February 2018 on these modified measures.

This research is being completed to assist the Cancer Institute NSW understand the impact of the multidisciplinary care measures on the NSW cancer system. It is also being completed as part of my Doctor of Public Health at the University of New South Wales.

This focus group today is going to explore how the measures have been interpreted and collected in each of your local health districts, discuss any changes that have happened in your cancer centres as a result of collecting data on these measures, explore any barriers and facilitators of data collection you have encountered locally, and identify any measures that could be developed for collection in the future.

There are some ground rules for the group today:

- Participation in this focus group is voluntary, please let the me know if you would like to leave the group at any time.
- All discussion that takes place within this group is confidential. Transcripts will be completed of the discussion with all information de-identified and confidentiality maintained.
- We are recording this session so can one person please speak at a time and we would like to ensure all participants have the opportunity to participate in the discussion.
- All responses are valid, there are no right or wrong answers.
- My role as facilitator is to help keep the discussion on track end ensure we are able to cover the main topics in the time allocated.

Each question will be put on PowerPoint slides to be projected in the room.

**Primary questions:**

Q1. I would like each of you to give a brief introduction of yourself and your experience and role with the multidisciplinary care measures. Has it facilitated other aspects of your role? what are the main barriers?

- Talk about examples, what has happened, asking them to tell a story
- A couple of minutes each, don’t be offended if I cut you off.

Q2. To what extent have processes been developed in your local health district to enable the regular collection of the multidisciplinary care measures?

- Outline the processes developed
- Have these processes been implemented?
- I would like to hear peoples thoughts on how/if this was influenced by the systems and infrastructure available in your local health district or cancer centre?
- To what extent has the process of collecting and reporting on the measures facilitated discussions about the documentation in multidisciplinary team meeting discussion and the use of data?

Q3. Could you describe what you and others in your local health district perceived to be the purpose of the measures?

- Did different understandings in your local health district of the purpose of the measures ever pose a barrier to or facilitate the collection and setting up of systems for automated collection of data?
- Did the refinement of the measures for FY2017/18 help you and your colleagues to better understand the purpose of the measures?
- How could the measures be further refined or developed?
- How could this improve the measurement of multidisciplinary care in NSW and the documentation and data collection process?

Q4. In what ways have you and your team used the reports the Cancer Institute NSW provided to each local health district?

- What would make the report more useful to you?
- What could be done differently?

**Summary question:**

Facilitator to give a short summary of the key questions and ideas that have emerged during the discussion

- Is anything missing from the summary of what was discussed here today?
- Has anything discussed surprised you?

**Final Question:**

The aim of this focus group today was to explore how the multidisciplinary care measures have been collected and used to support multidisciplinary team improvement in local health districts and cancer centres across NSW.

- Is there anything we haven’t discussed that you think is important towards this end?
- Are there any key ideas missing?

Invite participants to make contact with the moderator via phone or email to discuss further if there are other ideas or issues they would like to raise outside of the focus group.

**Probes/prompts (Avoid paraphrasing)**

Would you say more?

Tell us more about that

Could you give an example?

Is there anything else?

Does anyone have any different opinion about that?

Is there anything else?

I see people nodding their heads, tell me about it.

Could you explain further?

Sit with silences, don’t jump in too quickly
